# Effect of Using the Food Quotient as a Proxy of the Respiratory Quotient in the Calculation of Energy Expenditure by the Doubly Labeled Water Method in Older Adults

**DOI:** 10.1002/ajhb.70222

**Published:** 2026-02-25

**Authors:** Dennis Gustavo Alves de Mello, Luiz Antonio dos Anjos, Michele Novaes Ravelli, Eduardo Ferriolli, Karina Pfrimer

**Affiliations:** ^1^ Postgraduate Program in Nutrition and Metabolism, Ribeirão Preto Medical School University of São Paulo Ribeirão Preto Brazil; ^2^ Postgraduate Program in Food, Nutrition and Health, Institute of Nutrition Rio de Janeiro State University Rio de Janeiro Brazil; ^3^ Postgraduate Program in Nutrition Sciences, Laboratory of Nutritional and Functional Assessment, Department of Social Nutrition Fluminense Federal University Niterói Brazil; ^4^ Biotechnology Center & Nutritional Sciences University of Wisconsin‐Madison Madison Wisconsin USA; ^5^ Department of Clinical Medicine, Ribeirão Preto Medical School University of São Paulo Ribeirão Preto Brazil; ^6^ Division of Geriatrics, Department of Internal Medicine University of São Paulo Medical School São Paulo Brazil; ^7^ Postgraduate Program in Biotechnology University of Ribeirão Preto Ribeirão Preto Brazil; ^8^ Nutrition School University of Ribeirão Preto Ribeirão Preto Brazil

**Keywords:** doubly labeled water, food quotient, respiratory quotient, total energy expenditure

## Abstract

**Background and Aims:**

Accurate measurement of total energy expenditure (TEE) is critical for maintaining energy balance and body weight. This study aimed to analyze differences in TEE assessed by the doubly labeled water method (DLW‐TEE), using food quotient (FQ) derived from self‐reported 24‐h dietary recalls, respiratory quotient measured by indirect calorimetry (RQ‐IC), and usual respiratory quotient of 0.85 (RQ‐0.85) based on Western‐type diet intakes.

**Methods:**

Secondary analysis from a cross‐sectional study conducted in a sample of 41 independent (21 women) older people (≥ 60 years). FQ was obtained from three self‐reported 24‐h dietary recalls, RQ‐IC was measured after an overnight fast under resting conditions. Repeated measures analysis of variance was used to compare differences in DLW‐TEE calculated with FQ, RQ‐IC, and RQ‐0.85.

**Results:**

DLW‐TEE was significantly different between the three approaches (*p* = 0.025). The RQ approaches on DLW‐TEE did not differ significantly between sexes (*p* = 0.325). The overall mean DLW‐TEE RQ‐0.85 was 2253 (SD = 529, 95% CI: 2086, 2420) kcal/day, DLW‐TEE RQ‐IC was 2251 (SD = 541, 95% CI 2090, 2431) kcal/day, and DLW‐TEE FQ was 2208 (SD = 534, 95% CI 2039, 2376). DLW‐TEE calculated with FQ significantly reduced TEE compared to the mean DLW‐TEE with RQ‐0.85 values (ΔTEE −45 kcal/day, *p* < 0.001).

**Conclusion:**

Self‐reported dietary intake data may provide a more context‐specific estimate of the FQ than relying solely on RQ from indirect calorimetry or the fixed RQ of 0.85 in DLW‐based TEE calculations. Although the resulting differences in TEE are modest, they can lead to overestimation of energy requirements over time.

## 1 Introduction

Aging is a multifaceted process characterized by significant physiological and psychological changes, for which adequate nutrition is crucial for maintaining health (Amarya et al. 2015; Leslie and Hankey 2015). It is well‐documented that older adults experience a reduction in the functional capacity of their organs and tissues, resulting in a lower adaptive capacity to stress (Fedarko 2011). The lower functional capacity promotes dependency, reducing the performance of individuals in activities of daily living (Lino et al. 2008). This condition is associated with a higher prevalence of chronic noncommunicable diseases, associated with polypharmacy (Boscatto et al. 2013), directly affecting the quality of life and well‐being. These factors cause a chronic energy deficiency affecting (Legesse et al. 2019) nutritional status of these older people (Kemoun et al. 2022). Therefore, a better understanding and accurate estimation of daily energy expenditure and requirements is critical to minimizing negative energy balance in an attempt to mitigate the decline in the health status of older adults (Cooper et al. 2013; Hill et al. 2013).

Currently, the doubly labeled water (DLW) technique is the gold standard method for determining the total energy expenditure (TEE) under free‐living conditions (Pontzer et al. 2021). In this method, carbon dioxide production is converted to energy using an estimate of the respiratory quotient (RQ), the ratio of carbon dioxide production to oxygen consumption by the whole body (Gan et al. 2025). Usually, the value of 0.85 is adopted, which was established for a Western‐type diet and can vary depending on the energy contribution of the macronutrients and substrate oxidation (Black et al. 1986). The usual Western‐type diet has 11% of energy derived from protein, 38% from fat, and 6% from alcohol (Cole et al. 1990). The RQ could be measured by indirect calorimetry, or under energy balance conditions, the food quotient (FQ) can be used as a surrogate for the RQ. This study aimed to assess whether there are differences in TEE calculated using FQ obtained from self‐reported 24‐h dietary recalls, RQ measured after an overnight fast by indirect calorimetry (RQ‐IC), and TEE calculated using RQ of 0.85 (RQ‐0.85) in a sample of Brazilian older adults.

## 2 Methods

This study is a secondary analysis from a previous cross‐sectional study conducted by our research group at the Ribeirão Preto Medical School, University of São Paulo. The original study was conducted in two phases, and the methodology for participant recruitment has been described in detail elsewhere (Pfrimer et al. 2015). The study was approved by the local Human Research Ethics Committee (CAAE 68635923.6.0000.5440) and all participants signed an informed consent prior to participating. The primary outcome was TEE of older adults. A post hoc power analysis based on the observed effect size between adjusts and sample size showed a power of 64%. Participants were weighed in kilograms using a calibrated scale (Filizola S.A, ID 1500, Brazil) in the morning after an overnight fast. Height was measured using a wall‐mounted stadiometer. Body mass index (BMI) was calculated and classified according to the Pan American Health Organization's (OPAS 2002) recommended cutoffs for older people: underweight, ≤ 23; eutrophy, 23–28; overweight, 28–30; and obesity, ≥ 30 kg/m^2^.

Body composition was determined by dual‐energy x‐ray absorptiometry (DXA) (Hologic Inc., QDR 4500w, USA) at first phase of the study at Ribeirão Preto Medical School. DXA was calibrated daily by a phantom for mineral content and other parts. Free‐fat mass (FFM) was the sum of mineral and nonmineral content. Total body water (TBW) was calculated from FFM assuming a stable hydration constant of 73.2%.

The resting energy expenditure (REE) of the 41 participants was determined by indirect calorimetry (Vmax 29 series, Sensormedics, USA) in the second phase of study at Ribeirão Preto Medical School and before the DLW phase. Participants were instructed to abstain from alcohol for 48 h, exercise or drink coffee for 24 h, and fast overnight for 12 h before the test. The participants were allowed to drink only water during the fasting period. REE was measured early in the morning for 30 min with the participant lying supine in a quiet, low‐light environment, and ambient temperature at approximately 23°C. The rates of oxygen consumption (VO_2_) and carbon dioxide production (VCO_2_) were obtained over 30 min. The initial 5 min of the gas exchange values were not considered in the analyses. RQ was obtained from the ratio between VCO_2_ and VO_2_.

Self‐reported 24‐h dietary recalls were applied on initial day of DLW phase, Day 5, and Day 10 after dose administration of DLW, in home setting collection, by trained interviewers using with five‐step multiple‐pass method. Participants were asked about their beverage and food intake from the previous day, including time, occasion, and the amount consumed. Photos and drawings of utensils and common portions were used to estimate the amount consumed (Monteiro et al. 2007).

The Nutrition Data System (NDS) software (NCC, 2007, University of Minnesota—USA) was used to analyze the nutritional value of the self‐reported 24‐h dietary recalls. Additional chemical composition data of typical Brazilian foods were obtained from the Brazilian Composition Table of the Brazilian Institute of Geography and Statistics (Instituto Brasileiro de Geografia e Estatística 2011). The results obtained were attenuated by the method proposed by Iowa State University (Nusser et al. 1996) using the PC‐Side software (Iowa State University, 2003), reducing the distortion of consumption estimates. Considering the potential biases introduced by misreporting of dietary intake, under‐reporting and over‐reporting were assessed using the equation proposed by Bajunaid et al. (2025). This equation enables the identification of potentially erroneous or implausible self‐reported data, thereby helping to minimize the impact of misreported intake on the accuracy of our analysis.

The mean FQ was calculated from the nutritional values of the three self‐reported 24‐h dietary recalls using the following Equation (1) from Black et al. (1986).

(1) $$\text{Food quotient}\;\left(\mathrm{FQ}\right)=\left(p\times 0.81\right)+\left(f\times 0.71\right)+\left(c\times 1.00\right)+\left(a\times 0.67\right)$$

where *p*, *f*, *c*, and a correspond to the percentage of energy contribution of proteins, fats, carbohydrates, and alcohol. The energy contribution of each nutrient was calculated by multiplying the amount consumed (g) by its energy conversion factor, which is 4 kcal/g for proteins, 9 kcal/g for fats, 3.75 kcal/g for carbohydrates, and 7 kcal/g for alcohol (12). Total energy intake was calculated by summing the energy contribution of each nutrient. The percentage of energy contribution was calculated by dividing the energy contribution of each nutrient by the total energy intake.

The DLW method was used to measure the rate of CO_2_ production over a period of 10 days, which was subsequently used to calculate the TEE using the three different approaches. A baseline urine sample was collected before administering a dose of 2.0 g/kg of TBW of oxygen‐18 enriched to 10APE H_2_^18^O (YEH0011N, TAIYO NIPPON SANSO Corporation, JPN) and 0.12 g/kg of deuterium oxide enriched to 99.8APE ^2^H_2_O (617 385, Sigma‐Aldrich, USA). The dose was ingested through a straw to avoid losses at research facility of university on the second phase of the study. The dose container was rinsed twice with 50 mL of drinking water to ensure complete dose ingestion. Urine samples were collected 3, 4, and 5 h at research facility, and on 5 and 10 days after the ingested dose in a home visit. The samples were analyzed by isotope ratio mass spectrometry (Hydra 20‐20, Europa Scientific, Cheshire, England) at the Mass Spectrometry Laboratory of the Ribeirao Preto Medical School. All calculations were adjusted for the content of isotopes in drinking water. Isotope dilution spaces and elimination rate were calculated following the protocols recommended by Scrimgeour et al. (1993) and Schoeller et al. (1986) using the two‐point method Cole et al. (1990). The production of CO_2_ was calculated according to the equation reviewed by Speakman et al. (2021). TEE was calculated using the Equation (2) proposed by Weir (1949), considering the three approaches (RQ‐0.85, RQ‐IC, and FQ). Data obtained in MJ/day were converted to kcal/day using a conversion factor of 239.0057.

(2) $$\mathrm{TEE}\;\left(\frac{\mathrm{Mj}}{\mathrm{day}}\right)=\mathrm{rCO}2*\left(1.106+\left(\frac{3.94}{\mathrm{RQ}}\right)\right)*\left(\frac{4.184}{10^{3}}\right)$$

The normality of the data was assessed using the Shapiro–Wilk test. An independent sample *t*‐test was used to assess differences between men and women. Mann–Whitney *t*‐test was used for nonparametric variables. One‐sample *t*‐test was used to compare the values obtained for the RQ‐IC with the usual value of 0.85. Repeated measures analysis of variance (ANOVA) was used to compare differences in TEE between the three approaches to measure the TEE, and the Greenhouse–Geisser correction was applied when Mauchly's assumption of sphericity was violated, followed by the Bonferroni post hoc test. The inclusion of sex in the ANOVA allowed the evaluation of the within‐subject and between‐subject interaction effects. RStudio (Posit Software, PBC, Boston, MA, version 2023.12.1.402) was used for statistical analyses. All tests were two‐tailed. The significance level adopted was *p* < 0.05.

## 3 Results

Table 1 shows the demographic and anthropometric characteristics of the participants. The mean BMI and body fat percentage were significantly higher for women, who had significantly less FFM compared to men. Obesity was observed in 13 participants (32%), with the majority being women (10 participants, 77%). Eutrophy was observed in 17 participants (41%), with men representing 10 participants (59%). Underweight was observed in nine participants (21%), predominantly men (seven participants, 71%). Overweight was observed in four participants (10%), with an equal distribution between the sexes.

**TABLE 1:** Demographics and anthropometric characteristics of the 41 participants.

Characteristics	Women (*n* = 21)			Men (*n* = 20)			*p* ^a^
	Mean	SD	95% CI	Mean	SD	95% CI	
Age, years	67.4	3.6	65.7;69.0	67.7	4.4	69.7;65.6	0.80
Weight, kg	68.3	11.5	63.0;73.5	75.8	12.7	81.7;69.9	0.055
Height, cm	153.5	6.2	150.7;156.3	170.9	6.1	173.7;168.0	< 0.001
BMI, kg/m^2^	29.0	4.9	26.9;31.2	25.9	4.1	27.8;24.0	0.034
Fat, %	41.0	5.3	38.6;43.4	25.9	5.3	28.4;23.4	< 0.001
Free‐fat mass, kg	37.5	4.5	23.4;39.5	53.4	7.5	57.0;49.9	< 0.001

Abbreviations: 95% CI, 95% confidence interval; BMI, body mass index.

^a^ Student's *t*‐test.

Table 2 shows the mean dietary intake obtained from three self‐reported 24‐h dietary recalls, the percentage energy contribution for each macronutrient, the FQ obtained using Equation (1), and the RQ‐IC after an overnight fast under resting conditions (Table 2).

**TABLE 2:** Mean energy intake, percentage energy contribution for each macronutrient, food quotient from three self‐reported 24‐h dietary recalls, and respiratory quotient obtained by indirect calorimetry of the 41 participants.

	Women (*n* = 21)			Men (*n* = 20)			
	Mean	SD	95% CI	Mean	SD	95% CI	*p* ^a^
Energy intake, kcal/day	1551	716	1225; 1877	2284	481	2059; 2509	< 0.001
Carbohydrate, %	51.9	10.2	47.2; 56.5	45.8	7.5	42.3; 49.3	0.036
Protein, %	18.0	4.5	15.9; 20.0	17.8	3.9	16.0; 19.6	0.916
Fat, %	29.9	8.6	26.0; 33.8	31.8	6.4	28.8; 34.8	0.426
Alcohol, %	0.0^b^	0.80	0.1; 0.5^c^	2.9^b^	7.1	1.1; 7.8^c^	< 0.001^d^
FQ	0.883	0.027	0.871; 0.895	0.863	0.021	0.854; 0.873	0.013
RQ‐IC	0.849	0.055	0.824; 0.874	0.853	0.062	0.823; 0.882	0.855

Abbreviations: %, corresponds to the energy contribution of each macronutrient and alcohol in total energy intake; 95% CI, 95% confidence interval; FQ, food quotient from three self‐reported 24‐h dietary recalls; RQ‐IC, respiratory quotient by indirect calorimetry.

^a^ Student *t*‐test.

^b^ Median.

^c^ p25 and p75.

^d^ Mann–Whitney *t*‐test.

As shown in Table 2, the energy intake of women was 733 (SD = 192) kcal/day lower (*p* < 0.001) than men. Women reported significantly higher carbohydrate and lower alcohol intakes than men, leading to a significantly higher FQ compared to men. The overall mean FQ for 123 self‐reported 24‐h dietary recalls was 0.873 (SD = 0.021), with a coefficient of variation of 2.4%.

A one‐sample *t*‐test showed significant differences in FQ for both sexes, men (*p* = 0.009) and women (*p* < 0.001) compared to RQ‐0.85. No significant difference was found for RQ‐IC (*p* = 0.859) for men and women (*p* = 0.938) when compared to RQ‐0.85. According to the equation proposed by Bajunaid et al. 2025, six women (28.6%) and three men (15%), totalizing nine participants (22%), reported an energy intake below the 95% prediction interval and were classified as under‐reporters. These data were not excluded from the calculated RQ obtained from food consumption. We conducted a sensitivity analysis with and without the under‐reporters and showed no significant differences in mean or major changes in 95% CI TEE‐FQ values (see Supporting Information S1).

There was a significant difference in TEE between sexes (*p* = 0.018) and when RQ was adjusted with the approaches (*p* = 0.025). However, the interaction within subjects between RQ adjustment and sex was nonsignificant (*p* = 0.325), suggesting that the influence of RQ approaches on TEE did not differ substantially between men and women. The overall mean DLW‐TEE RQ‐0.85 was 2253 (SD = 529, 95% CI 2086, 2420) kcal/day, mean DLW‐TEE RQ‐IC was 2251 (SD = 541, 95% CI 2090, 2431) kcal/day, and DLW‐TEE FQ was 2208 (SD = 534, 95% CI 2039, 2376) kcal/day.

Bonferroni post hoc analyses revealed that TEE was lower in women compared to men, as expected (ΔTEE −386 kcal/day, *p* = 0.018). The mean DLW‐TEE was 2052 (95% CI 1832, 2273) kcal/day for women and 2438 (95% CI 2212, 2664) kcal/day for men. Using the FQ approach in DLW‐TEE calculations significantly reduced TEE compared to the mean RQ‐0.85 (ΔTEE −45, 95% CI −61, −30) kcal/day (*p* < 0.001). There were no significant differences in DLW‐TEE when the RQ‐IC was used in comparison to RQ‐0.85 (ΔTEE +7, 95% CI −47, 32) kcal/day (*p* = 1.0), nor when compared to the FQ obtained by three self‐reported 24‐h dietary recalls (ΔTEE −52, 95% CI −99, 7) kcal/day (*p* = 0.075). We examined which RQ approach produced the lowest TEE value within each participant. FQ adjust produced the lowest TEE value in 23 participants (56%), whereas RQ‐IC produced the lowest TEE value in 14 participants (35%) and RQ‐0.85 produced the lowest value in 4 participants (9%).

## 4 Discussion

FQ is a variable that can be used to estimate the oxidation of substrate mixtures obtained from the diet, an important factor when calculating the TEE with the DLW method (Hall et al. 2019). To date, no studies have investigated Brazilians FQ, especially of older people. In our current study, FQ was different from RQ obtained after an overnight fast under resting conditions by indirect calorimetry. Large energy imbalances, with a magnitude of 50% underfeeding or overfeeding of TEE, will alter RQ by 4%–11% (Elia and Livesey 1992). These imbalances can be detected by changes in the participant's body weight through the 10–20 days required by the DLW method (Elia and Livesey 1992).

Previous studies with the DLW method have demonstrated that a higher carbohydrate intake may lead to a reduction in TEE. In a randomized control trial, Ebbeling et al. (2018) reported that diets with 60% of total energy contribution from carbohydrates reduced TEE by 50 kcal/day, and a diet with 20% of energy contribution from carbohydrates increased TEE by 200 kcal/day. A linear trend was observed, indicating an increase of 52 kcal/day in TEE for every 10% decrease in carbohydrate contribution intake. In contrast, Hall et al. (2019) found a more modest increase of 50 kcal/day in TEE on a ketogenic diet with a 5% carbohydrate contribution compared to a standard diet of 50% carbohydrate contribution.

Although diet‐induced thermogenesis (DIT) is the smallest component of TEE, it could play a role in energy balance (Ebbeling et al. 2018). DIT is different for each nutrient, and reported values are 0%–3% for fat, 5%–10% for carbohydrates, 20%–30% for protein, and 10%–30% for alcohol (Westerterp 2004). The main determinants of DIT are the energy content, the protein, and alcohol contribution of the diet (Weststrate 1993) and body fatness (Segal et al. 1992). Additionally, participants with obesity tended to exhibit a lower thermic response compared to lean participants (Segal et al. 1992).

The FQ was calculated from three self‐reported 24‐h dietary recalls, which may have limited accuracy. Although the number of under‐reporters in our sample appears lower than in other studies (Bajunaid et al. 2025), this comparison is based on our perception and not on a formal statistical assessment. Importantly, misreporting in women represents a substantial proportion of the sample, which could introduce meaningful bias in our final results. Participants' characteristics are the main determinant of under‐reporting, but BMI and protein intake play an important role (Bajunaid et al. 2025). Higher body fatness, especially in women, is associated with a high proportion of under‐reporting (Pfrimer et al. 2015), resulting in differences in dietary energy intake and metabolizable energy of individual energy substrates (Elia and Livesey 1992). There was a strong positive relationship between the reported protein intake and under‐reporting (Bajunaid et al. 2025). It should also be noted that there is uncertainty in using indirect calorimetry to accurately assess the oxidation of energy substrates. There is a bias of approximately 5% above the true value in individuals at rest due to imprecision in the measurement of flow and fractional concentrations of oxygen and carbon dioxide (Elia and Livesey 1992).

RQ‐IC may not represent the actual RQ of the participants in this study because the measurement was conducted in a fasting state, and it was not adjusted for protein oxidation. A recent study showed a small difference (≅ +0.03) between the 24 h RQ (approximately 0.85), measured in whole‐body indirect calorimetry, and the sleeping RQ in a sample of 13 healthy young men (Ando et al. 2024). RQ‐IC in the present study was comparable to the adopted RQ for Western‐type diet used in most studies that apply the DLW method when 24 h RQ‐IC values are unknown. The use of the FQ as a proxy for RQ is a routine procedure to approximate RQ estimates in such cases. However, random and systematic errors may arise from this approximation approach. To minimize these errors, we adopted recommended procedures, including a standardized protocol, trained interviewers, and graduated photographic materials to reduce incorrect estimation of portion sizes. However, memory lapses can affect 24‐h dietary recalls due to failure to recall foods consumed or incorrect reporting of foods that were not consumed during the recalled day (Gibson et al. 2017). The use of equation proposed by Bajunaid et al. 2025, derived from DLW data, is essential for identifying the actual level of under‐reporters and could be used as quality control of 24‐h dietary recalls for FQ calculation, reducing biases.

From a practical perspective, dietary intake is already assessed in most DLW studies. In this context, deriving RQ from dietary intake represents a pragmatic approach, as it relies on data that are already collected and does not impose additional burden on participants. In contrast, measuring RQ via indirect calorimetry is more complex, costly, and logistically demanding, requiring calibrated equipment, trained personnel, and strict adherence to pre‐test standardization procedures. When dietary data are available and appropriately quality‐controlled, estimating RQ from dietary intake provides a favorable balance between feasibility and accuracy, while remaining low‐cost and less burdensome for participants.

Our study identified a statistical difference in TEE values when calculated using FQ compared to either RQ method. Although modest in magnitude, this difference may have meaningful implications for understanding gradual changes in body weight and body composition, and consequently, energy requirement estimations (Niclou et al. 2023). Persistent imbalances, even of this scale, can lead to substantial weight changes in the long term. For instance, Hall et al. (2011) demonstrated that a positive energy balance of just 24 kcal/day could eventually result in a weight gain of approximately 1 kg, with 50% of this gain occurring within the 1 year and 95% within 3 years. However, sustaining weight gain beyond this initial phase requires a dynamic adjustment in the energy balance, meaning that energy intake must progressively increase to compensate for the increase in energy expenditure associated with increased body weight or compensation for the energy gap (Prado‐Nóvoa et al. 2024).

Energy imbalance values can vary across populations due to differences in their dietary habits, physical activity levels, and metabolic factors. In China, an imbalance of 45 kcal/day is considered sufficient to promote weight gain in the majority of the population over the long term (Zhai et al. 2008). Predictive models that do not incorporate this gap are likely to overestimate energy needs, which may, in turn, lead to excessive dietary recommendations and hinder weight management efforts.

Applying equations derived from populations with different energy balance profiles, without appropriate adjustments for local factors, could yield unrealistic energy requirements. This oversight has the potential to undermine the effectiveness of nutritional interventions and public health policies aimed at addressing weight‐related challenges. As a result, integrating population‐specific energy balance and FQ data is crucial for improving the accuracy of predictive models, thereby enabling the development of more tailored and effective strategies.

## 5 Conclusion

Although the reduction in DLW‐TEE calculated with FQ is statistically significant compared to values derived from RQ‐IC or RQ‐0.85, the absolute difference is modest when relative to mean DLW‐TEE values. However, even small discrepancies can contribute to meaningful overestimations of energy requirement, particularly in long‐term energy balance studies. Incorporating plausible dietary intake data to estimate FQ may provide a more individualized and accurate approach than relying solely on RQ‐IC or the universal RQ‐0.85 adopted from a Western‐type diet.

## Author Contributions

**Dennis Gustavo Alves de Mello:** conceptualization, data curation, writing original draft, formal analysis, funding acquisition, writing – review and editing, and resources. **Karina Pfrimer:** conceptualization, methodology, investigation, writing original draft, formal analysis, funding acquisition, writing – review and editing, and resources. **Eduardo Ferriolli:** conceptualization, methodology, investigation, and resources. **Luiz Antonio dos Anjos:** writing – review and editing. **Michele Novaes Ravelli:** formal analysis and writing – review and editing.

## Funding

This work was supported by Fundação de Amparo à Pesquisa do Estado de São Paulo (Grant numbers 2023/09439‐7 and 07/50150‐8).

## Conflicts of Interest

The authors declare no conflicts of interest.

## Supporting information


**Data S1:** ajhb70222‐sup‐0001‐Supinfo.pdf.

## Data Availability

The data that support the findings of this study are available on request from the corresponding author. The data are not publicly available due to privacy or ethical restrictions.
