# Supplementary material for: Effect of Using the Food Quotient as a Proxy of the Respiratory Quotient in the Calculation of Energy Expenditure by the Doubly Labeled Water Method in Older Adults
Source: Am J Hum Biol. 2026 Feb 25;38(3):e70222. doi: 10.1002/ajhb.70222 (PMC12935522; doi:10.1002/ajhb.70222)
Supplement: Supplementary file 1 — Data S1: ajhb70222‐sup‐0001‐Supinfo.pdf. [file AJHB-38-e70222-s001.pdf]

Total energy expenditure with under-reporters

Descriptives

	N	Missing	Mean	95% Confidence Interval		Median	SD
				Lower	Upper		
tee-rq-0.85	41	0	2253	2086	2420	2210	529
tee-rq-ic	41	0	2261	2090	2431	2203	541
tee-fq	41	0	2208	2039	2376	2100	534

Note. The CI of the mean assumes sample means follow a t-distribution with N - 1 degrees of freedom

Total energy expenditure without 9 under-reporters

Descriptives

				95% Confidence Interval			
	N	Missing	Mean	Lower	Upper	Median	SD
tee-rq-0.85	32	9	2254	2049	2460	2230	570
tee-rq-ic	32	9	2261	2046	2475	2189	595
tee-fq	32	9	2216	2009	2424	2185	575

Note. The CI of the mean assumes sample means follow a t-distribution with N - 1 degrees of freedom

Paired Samples T-Test

Paired Samples T-Test

			statistic	df	p	Mean difference	SE difference	95% Confidence Interval	
								Lower	Upper
tee-rq-0.85	tee-rq-0.85-withouth-under-report	Student's t	-0.0944	31.0	0.925	-13.1	138	-295	269
tee-rq-ic	tee-rq-ic-withouth-under-report	Student's t	-0.1806	31.0	0.858	-25.3	140	-311	260
tee-fq	tee-fq-ur-withouth-under-report	Student's t	-0.1309	31.0	0.897	-18.3	140	-303	267

Note. H_a $\mu_{\text{Measure 1 - Measure 2}} \neq 0$

Normality Test (Shapiro-Wilk)

			W	p
tee-rq-0.85	-	tee-rq-0.85-withouth-under-report	0.977	0.705
tee-rq-ic	-	tee-rq-ic-withouth-under-report	0.985	0.925
tee-fq	-	tee-fq-ur-withouth-under-report	0.974	0.613

Note. A low p-value suggests a violation of the assumption of normality
